# A Macaque Model of Mesial Temporal Lobe Epilepsy Induced by Unilateral Intrahippocampal Injection of Kainic Acid

**DOI:** 10.1371/journal.pone.0072336

**Published:** 2013-08-26

**Authors:** Ning Chen, Chong Liu, Na Yan, Wei Hu, Jian-guo Zhang, Yan Ge, Fan-gang Meng

**Affiliations:** 1 Department of Neurosurgery, Beijing Tiantan Hospital, Capital Medical University, Beijing, China; 2 Beijing Neurosurgical Institute, Capital Medical University, Beijing, China; 3 School of Public Health and Family Medicine, Capital Medical University, Beijing, China; 4 Department of Neurology, Mayo Clinic, Rochester, Minnesota, United States of America; Northwestern University Feinberg School of Medicine, United States of America

## Abstract

**Objective:**

In order to better investigate the cause/effect relationships of human mesial temporal lobe epilepsy (mTLE), we hereby describe a new non-human primate model of mTLE.

**Methods:**

Ten macaques were studied and divided into 2 groups: saline control group (n = 4) and kainic acid (KA) injection group (n = 6). All macaques were implanted bilaterally with subdural electrodes over temporal cortex and depth electrodes in CA3 hippocampal region. KA was stereotaxically injected into the right hippocampus of macaques. All animals were monitored by video and electrocorticography (ECoG) to assess status epilepticus (SE) and subsequent spontaneous recurrent seizures (SRS). Additionally, in order to evaluate brain injury produced by SE or SRS, we used both neuroimaging, including magnetic resonance image (MRI) & magnetic resonance spectroscopy (MRS), and histological pathology, including Nissl stainning and glial fibrillary acid protein (GFAP) immunostaining.

**Results:**

The typical seizures were observed in the KA-injected animal model. Hippocampal sclerosis could be found by MRI & MRS. Hematoxylin and eosin (H&E) staining and GFAP immunostaining showed neuronal loss, proliferation of glial cells, formation of glial scars, and hippocampal atrophy. Electron microscopic analysis of hippocampal tissues revealed neuronal pyknosis, partial ribosome depolymerization, an abnormal reduction in rough endoplasmic reticulum size, expansion of Golgi vesicles and swollen star-shaped cells. Furthermore, we reported that KA was able to induce SE followed by SRS after a variable period of time. Similar to human mTLE, brain damage is confined to the hippocampus. Accordingly, hippocampal volume is in positive correlations with the neuronal cells count in the CA3, especially the ratio of neuron/glial cell.

**Conclusions:**

The results suggest that a model of mTLE can be developed in macaques by intra-hippocampal injection of KA. Brain damage is confined to the hippocampus which is similar to the human mTLE. The hippocampal volume correlates with the extension of the hippocampal damage.

## Introduction

Mesial temporal lobe epilepsy (mTLE) with hippocampal sclerosis is one of the most common forms of drug-resistant partial epilepsy in humans [1]. Spontaneous seizures in mTLE are believed to be preceded by a precipitating event, such as brain trauma, cerebral infection, status epilepticus (SE), genetic factors or anoxic brain episodes. These early injuries may trigger neurobiological changes that result in a chronic epileptic condition clinically characterized by partial seizures with occasional secondary generalization [2]. mTLE is characterized by unilateral hippocampal sclerosis without consistent and reproducible alteration of other brain structures [2]–[4]. Hippocampal sclerosis includes extensive neuron loss in CA1 and CA3 sectors and in the hilus of dentate gyrus (DG) [5], [6]. Furthermore, clinicopathologic features of mTLE can be reproduced in experimental animals to some extent. Intraperitoneal injection of proconsulsive drugs, such as pilocarpine and kainic acid (KA), is the most widely used procedure to induce mTLE in rodents [7]–[10]. However, these systematic drug-induced models have been criticized recently, because they present with features that are not typical of human mTLE, such as bilateral hippocampal alterations, extensive damage in brain regions exceeding the temporal lobe, and the occurrence of generalized tonic-clonic seizures [4]. KA is structurally related to the excitatory neurotransmitter glutamate, and can evoke hippocampal epileptic form activity. For adult rodents, single injection of KA into the dorsal hippocampus can reproduce more alterations which are similar to been observed in human mTLE [11]–[14]. Unlike systemic pilocarpine and KA models, structural alterations are mainly ipsilateral to the injected dorsal hippocampus in local intrahippocampal KA models.

It has been reported that the anatomical and physiological differences in the hippocampus between rodents and primates are often cited as an explanation of the discrepancies between rodent epilepsy models and human epilepsies. However, nonhuman primates, like macaques, are closely related to humans in terms of genetics, physiological functions and biochemical metabolisms, and have been widely used as models for human clinical conditions. Over the past decades, several non-human primate epilepsy animal models have been developed, and numerous agents including penicillin, aluminum hydroxide, bicuculline methiodide, domoic acid, pentylenetetrazol, and coriaria lactone have been applied. However, these models have limitations to some extent, mainly because they are not stable or the mechanisms involved are not clear [15]–[21]. Therefore, the aim of this study is to induce epilepsy in a macaque model with KA unilateral intra-hippocampal injection, which helps to evaluate the epileptogenesis, and observe the behavioral manifestations, alteration of magnetic resonance image (MRI) & magnetic resonance spectroscopy (MRS), histological pathology with glial fibrillary acid protein (GFAP) immunostaining of the macaque epilepsy model.

## Materials and Methods

### Animals

Ten young male macaques (3–6 years old; 7.5–8.5 kg) were used in this study. All animals were provided by the Experimental Animal Center of Military Medical Sciences, China. The macaques were maintained in individual cages in a controlled environment of 120 cm (H)×80 cm (W)×75 cm (D) with both lateral walls of organic glass. The front, roof, and back walls were made of organic glass, whereas the bottom of the cage was made of metal net. The room was maintained at constant temperature: 22–25°C; humidity: 60–70% and illuminated on a 12/12 h light: dark cycles with lights on from 7∶00 AM to 7∶00 PM. Animals had free access to food and tap water. A mixture of fruits and a nutritional complement were given at 8∶00 AM and 4∶00 PM. The care and handling of animals were conducted in compliance with the Chinese Animal Welfare Act, the Guidance for Animal Experimentation of Capital Medical University, and Beijng guidelines for the care and use of laboratory animals. It has been approved by the Ethics Committee of the Capital Medical University affiliated Beijing Neurosurgical Institute (Process NO. 20120619). Moreover, efforts were made to minimize the number of studied animals and their sufferings.

### Electrode Implantation for Electrocorticography

**Figure 1:**
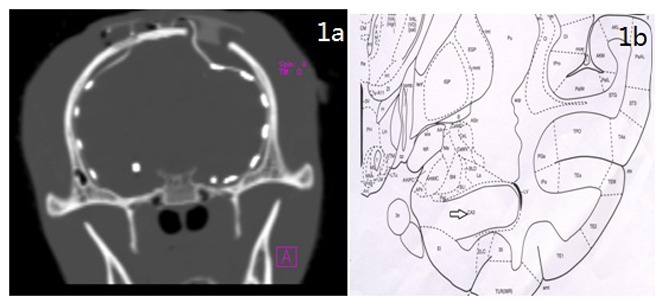
The location of recording electrodes and KA injection. a: CT one day after implantion of recording electrodes; b: The location of KA injection in the CA3 zone of the hippocampus. The schematic diagram was adapted from the Rhesus Monkey Brain in Stereotaxic Coordinates.

The macaques were sedated with ketamine hydrochloride (10 mg/kg, intramuscular; ShuangHe Pharmaceutical, Beijing, China). Each macaque was positioned in a stereotaxic frame (David Kopf Instruments, California, USA) with the orbitomeatal line parallel to the surface of the table. Deep recording electrodes (Beike, Beijing, China) were implanted in CA3 regions of bilateral hippocampus (stereotaxic coordinates: anteroposterior 8.1 mm, mediolateral ±12 mm, dorsoventra −35.3 mm relative to dura) and subdural recording electrodes were stereotaxically implanted into the temporal cortex of macaques by referring to the Rhesus Monkey Brain in Stereotaxic Coordinates [22], whereas indifferent and ground electrodes were implanted into the bone above cerebellum. Computed tomography (CT) scanning (GE, Fairfield, USA) was performed to check the location of the recording electrodes approximately 1 day post-operation at the Beijing Tiantan Hospital (Fig. 1a). To avoid postoperative infection, all macaques received gentamicin injection (5 mg/kg, intramuscular; Anyang Kyushu Pharmaceutical, China) for 3 days.

### Intrahippocampal KA Injection

The macaques were kept in individual cages for 1 week prior to drug injection. A camera (Canon, Tokyo, Japan) was positioned in front of the cage to record animals’ behaviors. Ten macaques were randomly divided into the KA group (n = 6) and the saline control group (n = 4). KA (2 µg/µl/kg; Sigma Chemical Co., St. Louis, MO, USA) was stereotaxically injected into the right hippocampus (stereotaxic coordinates: anteroposterior 8.55 mm, mediolateral +12 mm,dorsoventral −35.3 mm relative to dura according to the Rhesus Monkey Brain in Stereotaxic Coordinates [22]; Fig. 1b). The control macaques underwent the same procedure while KA was replaced by normal saline in an equivalent volume.

### Video- electrocorticography (ECoG) Recordings

Before KA injection, the macaques were monitored with ECoG for 6 h (baseline). The animals were monitored with video-ECoG for 6 h/day for 7 consecutive days after KA injection. After the acute post-KA phase, 6 h/day, 3 days video-ECoG recordings were performed every other week for no less than 12 weeks. Acute seizures were defined as seizures occurring within 24 h after KA injection.

The seizure intensities were classified from I to V according to the modified Racine scale [16], [23] as Table 1. The seizure behaviors, the latency from injection to seizure onset and the seizure duration were recorded and quantified after every KA injection.

**Table 1:** Seizure severity indexes.

Class	Behavior
I	Facial automatisms, including salivation, “mouth cleaning-like” behavior, and tongue automatisms
II	Facial movements, head clonus, and head shakes
III	Forelimb clonus
IV	Bilateral forelimb clonus, rearing, arched body posture, Straubtail
V	Generalized clonic seizures and postural impairment

### MRI & MRS Analysis

MRI *&* MRS were performed in the macaques approximately 3 months post-operation at the Beijing Neurosurgical Institute. The images were acquired from animals, already perfused, using a 3.0 T MRI scanner (Simenzi, Berlin, German). The acquisition protocol had been previously tested in a normal control animal to standardize the method. The acquisition protocol was as the following: field of view (FOV) = 150×180 mm^2^; matrix = 259×384; time of repetition (TR) = 4500 ms; time of echo (TE) = 84 ms; thickness of slices = 3.0 mm; distance between slices = 3 mm; total acquisition time = 2 h and 30 min. Volumetric studies were performed using a T1-weighted 3-D fast gradient-echo sequence with inversion preparation pulse and the following parameters: TR, 3000 ms; TE, 46 ms; FOV, 256×256; matrix, 512×512; distance between slices = 1 mm. Each acquisition was transferred to a Sparc Station 5 (Sun Microsystems, Mountain View, CA, USA) and analyzed using Easy Vision CT/MR software (Release 2; Philips Medical Systems, Eindhoven, the Netherlands). The data were reformatted in the tilted coronal plane perpendicular to the long axis of the hippocampus. Each hippocampal slice, 1 mm in thickness, was then measured. A 3-D contour was derived, from which the volumes of the right and left hippocampal formation were calculated. The boundaries of the hippocampal formation were outlined manually [24]. ^1^H-MR spectra were obtained from a multiple voxel placed over the hippocampus. The voxel position was determined from multiple slice echo planar images: FOV = 150×180 mm^2^; matrix = 259×384; TR = 4500 ms; TE = 84 ms; thickness of slices = 3.0 mm.

### Fixation and Processing of Tissue

All macaques were sacrificed three months after the KA injection. At the time of sacrifice, all macaques were deeply anesthetized with ketamine (10 mg/kg, intramuscular) and fixed on the operating table, and then transcardially perfused with 0.9% saline solution followed by 4% paraformaldehyde in 0.1 M sodium phosphate buffer, pH 7.4. Brains were removed from the skull, and the ventrolateral portion of the temporal cortex, including the hippocampus was dissected out. Each hippocampus was cut into 1.5 mm thick slabs, transverse to the rostrocaudal axis. Slabs were then postfixed in the paraformaldehyde/PB perfusion solution for 24 h.

### Hematoxylin and Eosin (H&E) Staining and Electron Microscopy

For H&E staining, 5 µm sections were prepared from formalin-fixed, paraffin-embedded samples. Samples were post-fixed in 2.5% glutaraldehyde for 3 h and then in a solution containing 1% osmium tetroxide, pH 7.3, at room temperature for 1 h. For electron microscopy the samples were dehydrated in a graded alcohol series and embedded in epoxy resin. Thin sections (70 nm) were cut and viewed using an electron microscope (Hitachi H-7650, Tokyo, Japan).

### Nissl Staining

Tissues were post-fixed for 24 h and then placed for 24 h in 30% sucrose in 0.1 M phosphate buffer. To identify the cytoarchitectonic boundaries, the distribution and severity of neuronal damage, coronal sections (5 µm) underwent Nissl staining with toluidine blue. Severity of neuronal damage in a section was semiquantitatively assessed by a grading system similar to the methods that previously described by Halonen et al. [25], Cilio et al. [26] and Brandt et al. [27] as follows: score 0, no obvious damage; score1, apparent alteration of morphology, but no unambiguous lesion; score 2, clear-cut lesions involving 20–50% of neurons; and score 3, clear-cut lesions involving>50% of neurons. In this respect, it is important to note that neuronal loss must exceed 15% to 20% before it is reliably detected by visual inspection [28]. Visual assessment was conducted blindly with respect to the treatment status of the animal. Assessment was performed in hilar regions, CA1, CA3, subiculum, temporal cortex, frontal cortex, entorhinal cortex, hypothalamus, thalamus and anterior hypothalamus in each animal.

### Immunohistochemistry

Immunohistochemistry for GFAP-positive cells (astrocyte markers) was performed using the ABC method. Coronal brain sections from KA macaques and control macaques were incubated overnight with primary polyclonal antibody against GFAP (1∶2000; DakoCytomation) followed by incubation with the appropriate secondary biotinylated antibody (1∶600; Vector Laboratories; anti-rabbit). Briefly, coronal sections (5 µm) were rinsed in potassium PBS, treated with 3% H_2_0_2_, and incubated overnight with 0.1% TritonX-100 plus PBS solution and primary antibody. Sections were rinsed and incubated in avidin-biotin complex (Elite ABC kit, Vector laboratories), and developed with diaminobenzidine. Sections were analyzed by conventional microscopy. GFAP-stained sections were quantitatively assessed as follows: score 0: no obvious expression, score 1: obvious expression involving 10–25% of the region, score 2: obvious expression involving 40–60% of the region, and score 3: >80% the expression in the regions of interest. Assessment was performed in hilar regions, CA1, CA3, subiculum, temporal cortex, frontal cortex, entorhinal cortex, hypothalamus, thalamus and anterior hypothalamus in each animal.

### Statistical Analysis

The statistical processing system used was SPSS 13.0 for Windows (SPSS, Chicago, IL, USA). Data were analyzed using a Student’s t-test and analysis of variance (ANOVA) followed by a Tukey-Kramer post hoc test. Simple correlations between MR variables (hippocampal volumes) and pathologic variables (neuron cell counts and glial cell counts) were performed using linear regression analysis. Pearson’s correlation coefficient (r) and F values were indicated. Data were presented as the mean± standard deviation (SD). *P*<0.05 was considered statistically significant.

## Results

### Acute Status Epileptic after Intrahippocampal KA Injection

**Figure 2:**
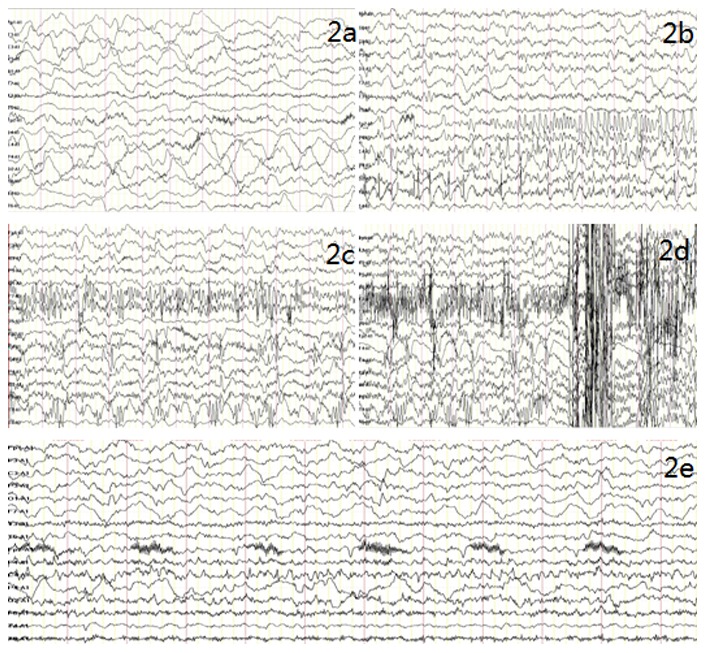
EEG recordings obtained after KA administration. The locations of the recording electrodes: Fp1, F3, C3,P3, 01 and F7 located in the left cortex zone; T3 located in the left hippocampus; T5 located in the left temporal lobe; FP2, F4, C4, P4, 02 and F8 located in the right cortex zone; T4 located in the right hippocampus; T6 located in the right temporal lobe. a: Dominated by slow waves and low amplitude fast waves; b: The right hippocampus first demonstrated spikes discharges that then were transmitted to the right cortex, followed by explosive spike rhythms in the right cortex; c: The left hippocampus demonstrates spike wave rhythms; d: Spine wave rhythms are shown; e: EEG after attack, dominated by slow waves and low amplitude fast waves.

The animals with accurate injection sites as confirmed by MRI were used. All KA animals exhibited seizure attack within (11.90±2.66) minutes (mean ± SEM). Additionally the KA induced acute behavioral effects, including salivation, vomiting, head shakes, tongue automatisms, “mouth cleaning-like” behavior and highly frequent orofacial movements, were observed at a similar latency (ranging from 10 to 20 minutes after KA injection) in these models. The beginning of SE was characterized by convulsive behavior stage IV. During SE, the KA macaque showed firstly forelimb clonus, afterwards bilateral forelimb clonus, rearing, arched body posture, straub tail, ultimately generalized clonic seizures. The latency for the beginning of SE was 15.6±2.0 min (n = 6). The duration varied from 1 h to 1.5 h (1.2±0.4 h). As expected, no such events were observed in saline-injected control group. In 6 macaques with KA injection, initially, ictal EEG event was observed in the treated hippocampus; then ictal activity could propagate from the onset site to other recording electrodes. The first seizure activity initiated in the hippocampus ipsilateral to KA injection subsequently spreaded to the ipsilateral neocortex, then to the contralateral hippocampus, and finally to the contralateral neocortex. The most frequent seizure pattern observed during the initial phase of the acute SE (n = 6) was characterized by low amplitude, high frequency spikes (16–18 Hz, 100–200 uV) at seizure onset, followed by dissemination of high amplitude, high frequency spikes(16–18 Hz, 300–500 uV) that subsequently organized in burst of high amplitude, and high frequency spikes(16–18 Hz, 300–500 uV) (Fig. 2).

### Spontaneous Seizures Develop after SE

In order to assess the development of SRS after the events of SE, we performed continuous Video-ECoG recording for several weeks. Specifically, SRS were observed in all KA models over the observation period of 3 months. The latency for SRS after SE varied from a few days (4 days, n = 2; 6 days, n = 1; 7 days, n = 1) to several weeks (3 weeks, n = 2). In addition, SRS frequency was higher in the initial weeks with a gradual reduction in the following weeks (Fig. S2). To further characterize the SRS, we evalutated the ECoG pattern and the behavioral manifestation. Tonic immobility was observed during the SRS, though, we did not notice the convulsive SE. The following nonictal (interictal) ECoG patterns were observed in 6 animals:(1) 3–8 Hz frequency, amplitude 200–500 µV, θ Wave and 10–14 Hz, band 100–200 µV, low amplitude rapid rhythm; (2) dissemination of spike and slow waves, frequency of 16–18 Hz, band 300–500 µV; (3) dominated by slow-waves, frequency of 2–4 Hz, band 200–400 µV. Notably, these patterns have never been observed during ECOG monitoring prior to KA treatment. One or more of those interictal patterns was identified in all studied KA models with chronic seizure. However the ictal patterns were not observed in the chronic phase.

### MRI & MRS

MRI and MRS were performed in all ten macaques. The MRI and MRS images were interpreted through “Double Blind Experiment” by two experienced radiologists respectively and a consensus reading was reached when the two radiologists reach a same interpretation no matter if they differed in their initial readings.

**Figure 3:**
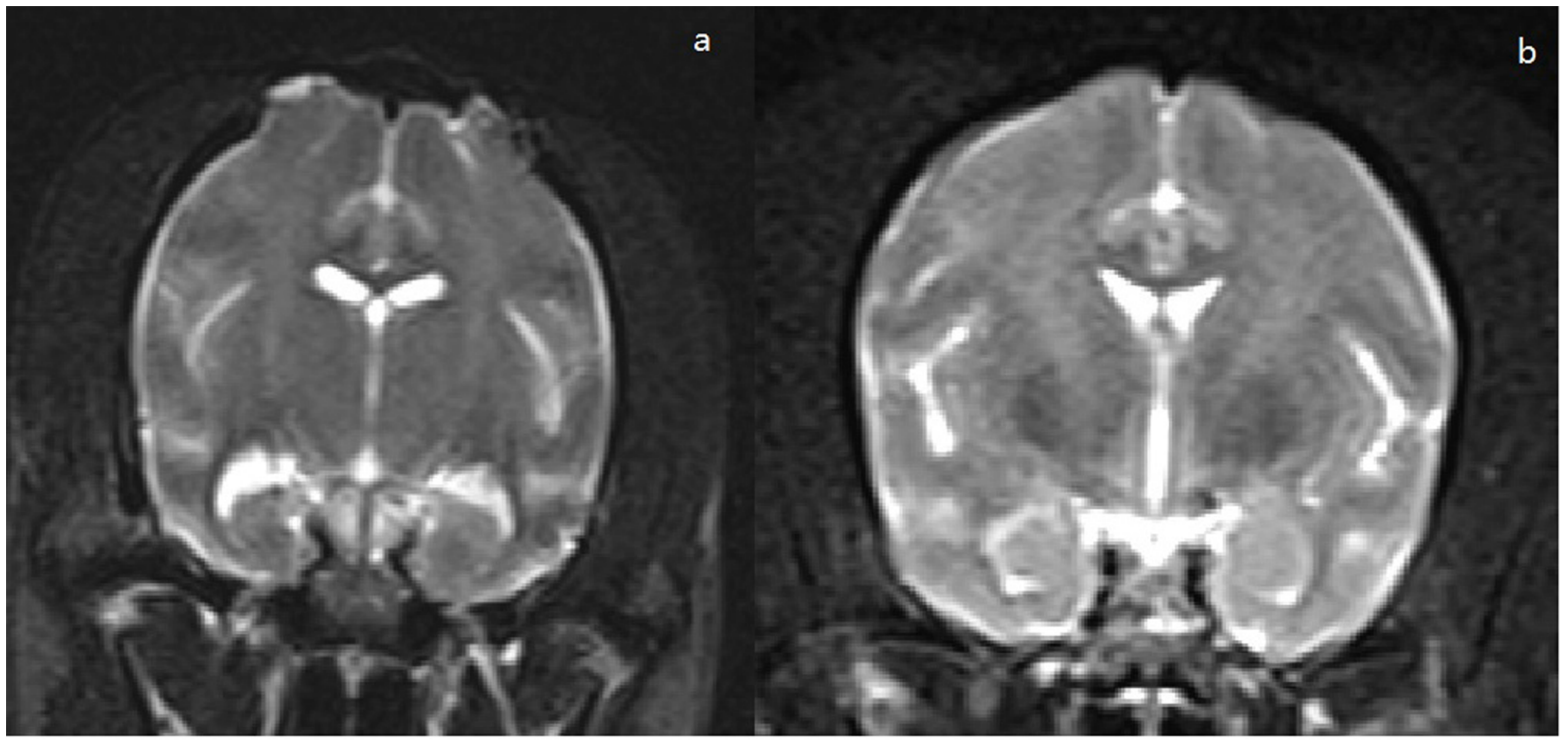
MRI features of macaque brains 3 months after drug injection. a: Coronal MRI in T2 sequence of a KA animal at 3.0 T; b: Coronal MRI in T2 sequence of a control animal at 3.0 T.

Fig. 3 shows the coronal T_2_ weighted images of the KA-injected macaques and the control macaque, respectively. Table 2 shows the MRI finding in the ten macaques. Comparing to control group, we found a significant reduction in ipsilateral hippocampal volume of KA group (*P*<0.05), however, no significant hippocampal volume reduction was found in the contralateral hippocampus of KA group (*P*>0.05, Fig. S1). Additionally, all 6 KA-injected macaques had hippocampal size decreasement (HSD); 5 had ipsilateral hippocampal signal increase (HIS) and 4 had temporal mandibular horn enlargement (TMHE). Notably, neither the contralateral hippocampus of KA-injected macaques nor the ipsilateral hippocampus of control macaques revealed the abnormality.

**Table 2:** MRI findings of hippocampus in KA group and Control group.

	N0.	Side	HSI	HSD	TMHE	HV(mm^3^)
KA-injectedMacaques(n = 6)	1	R	1	1	1	1565
		L	0	0	0	2793
	2	R	1	1	0	1948
		L	0	0	0	2752
	3	R	0	1	0	2061
		L	0	0	0	2740
	4	R	1	1	1	1706
		L	0	0	0	2641
	5	R	1	1	1	1693
		L	0	0	0	2694
	6	R	1	1	1	1801
		L	0	0	0	2714
ControlMacaques(n = 4)	7	R	0	0	0	2814
	8	R	0	0	0	2877
	9	R	0	0	0	2775
	10	R	0	0	0	2917

HSI = hippocampal signal increase; HSD = hippocampal size decrease; HV = hippocampal volume; TMHE = temporal mandibular horn enlargement; 0 = normal; 1 = abnormal.

**Figure 4:**
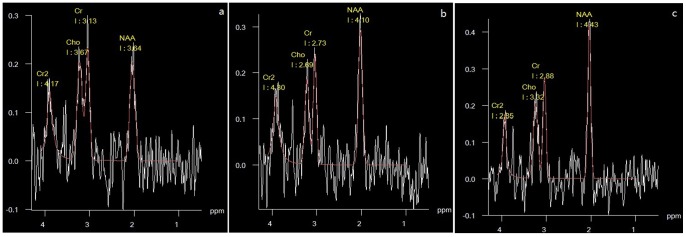
MRS features of macaque brains 3 months after drug injection. a: MRS of the ipsilateral hippocampus of a KA animal showed that NAA peaks at 3.64, Cho peaks at 3.13, and Cr peaks at 3.67 at 3.0 T; b: MRS of contralateral hippocampus of a KA animal showed that NAA peaks at 4.10, Cho peaks at 2.73, and Cr peaks at 2.60 at 3.0 T. c: MRS of right hippocampus of a control animal showed that NAA peaks at 4.43, Cho peaks at 2.88, and Cr peaks at 3.32 at 3.0 T.

Fig. 4 shows a typical edited ^1^H MR spectrum of a control and KA animal (12 weeks after injection) with all the important metabolites indicated. The metabolite concentrations obtained from these spectra are shown in Table 3. When comparing controls and KA animals at the same time points, the N-acetylaspartate (NAA) concentration was decreased by 34% (*P*<0.05) after SE, whereas choline-containing compounds (Cho) were increased (13% at 12 weeks, *P*<0.05) and creatine (Cr) was elevated (11%) at 12 weeks (*P*<0.05). The NAA/Cr ratios in the ipsilateral hippocampus declined significantly (35%, *P*<0.05; 36%, *P*<0.05) compared with the contralateral hippocampus and the hippocampus of the control group, respectively. Also the NAA/Cho ratios in the ipsilateral hippocampus declined significantly (35%, *P*<0.05; 38%, *P*<0.05) compared with the contralateral hippocampus and the hippocampus of the control group, respectively.

**Table 3:** Metabolite concentrations for control and KA macaques 3 months after the administration of KA.

KA macaques(n = 6)			Control group(n = 4)
	Ipsilateral	Contralateral	
NAA	3.07±0.64^*&^	4.23±0.27	4.47±0.34
Cho	3.57±0.63^*&^	3.10±0.17	3. 16±0.39
Cr	3.45±0.51^*&^	3.12±0.21	3.10±0.29
NAA/Cho	0.87±0.18^*&^	1.34±0.17	1.35±0.16
NAA/Cr	0.89±0.18^*&^	1.37±0.15	1.44±0.07

Mean ± standard deviation of NAA, Cho, Cr, NAA/Cho and NAA/Cr ratios for the ipsilateral and contralateral hippocampus in KA macaques and for both hippocampus in contral groups. Ipsilateral side vs. contralateral side: *P<0.05; Ipsilateral side vs. control groups; &*P*<0.05.

### Morphological Changes in the Hippocampus

**Figure 5:**
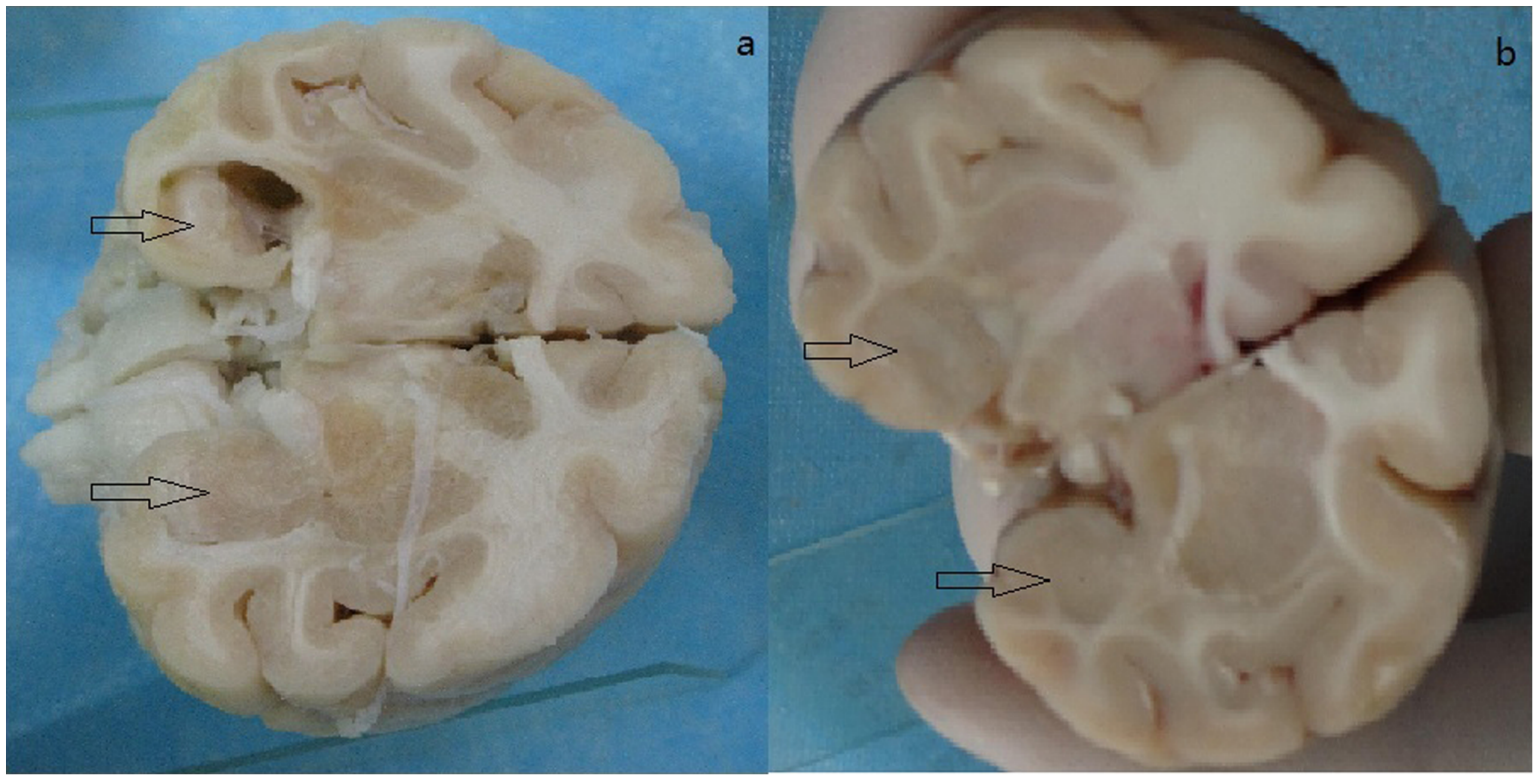
Observation of the specimens. a: hippocampus of KA macaques; b: hippocampus of control macaques.

**Figure 6:**
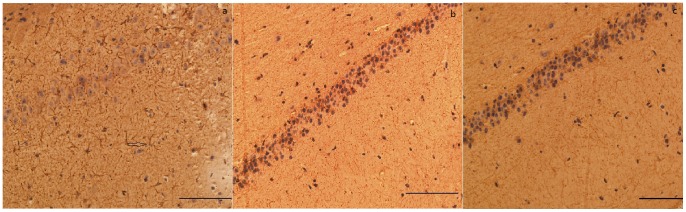
Strutural alternation in the chronic phase after intrahippocampal injection of KA. Sections with immunostained with antibodies against GFAP are illustrated. a:ipsilateral hippocampus of KA-injected (magnification ×200); b:contralateral hippocampus of KA-injected (magnification ×200); c: ipsilateral hippocampus of saline-injected (magnification ×200).

In line with the MRI findings, all KA treated models exhibited significant morphological changes. The hippocampus ipsilateral to the infusion was smaller than the contralateral hippocampus (Fig. 5). H&E staining of samples from the 6 KA-injected macaques revealed neuronal loss, organizational structure disorders in the hippocampal CA3 area, abnormal cell morphology. This extensive cell loss was accompanied by gliosis and microglial proliferation in the ipsilateral hippocampus. Immunohistochemistry for GFAP, the astrocytic marker, revealed its overexpression in the ipsilateral hippocamal CA3 region of the KA-injected macaques (Table S1). Moreover, the normal uniform distribution of astrocytes in these regions was replaced by a dense network of coarse glial fibers (reactive gliosis). However, neither KA treated group nor control group revealed any obvious neuronal cell loss or other morphological signs of damage (Fig. 6).

**Figure 7:**
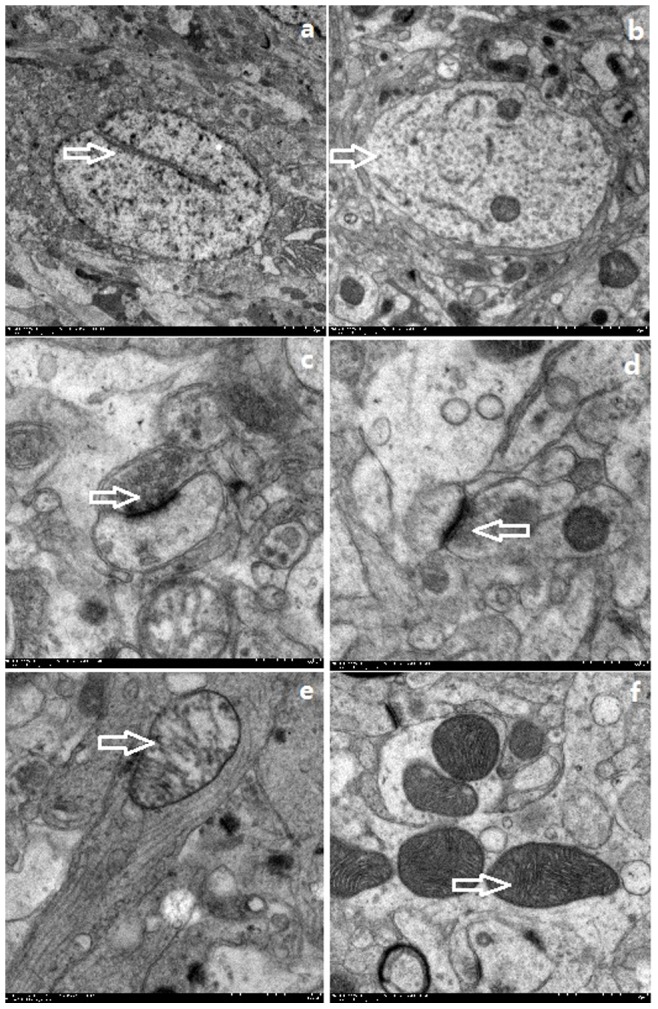
Electron microscopy of macaques. a: Nucleolemma introcession from the hippocampus of a KA macaque (magnification, 12,000×); b: Normal neuron from the hippocampus of control macaque(magnification; 25,000×); c: Presynaptic neurotransmitter increase from the hippocampus of a TLE macaque, (magnification; 80,000×); d: Normal presynaptic neurotransmitter in the hippocampus of control macaque(magnification, 80,000×) e: Disordered mitochondrial structure in the hippocampus of KA macaque, (magnification; 50,000×); f: Normal mitochondrial structure in the hippocampus of control macaque(magnificantion; 50,000).

Electron microscopic analysis of ipsilateral hippocampal tissues from the six macaques injected with KA demonstrated neuronal nucleolemma introcession and karyopycnosis, partial ribosome depolymerization, mitochondria with the abnormal appearance including swelling, disorganization and reduction or vanish of the crista, mild expansion of rough endoplasmic reticulum, expansion of Golgi vesicles, presynaptic excitability neurotransmitter increase, deposition of lipofuscin, and astrocytes cytoplasm swelling. The control hippocampal tissues gave evidence of normal structure (Fig. 7).

To investigate the effect of SRS on other brain regions induced by KA in macaquses, we evaluated neuronal damage in several brain areas, mainly in the limbic structures, by method of Nissl staining. The results are summarized in the Table S2. We did not observe cell loss in the hilar regions, CA1, subiculum, temporal cortex, frontal cortex, entorhinal cortex, hypothalamus, thalamus or anterior hypothalamus in either group of animals. However, neuronal loss was observed in the CA3 region of the ipsilateral hippocampus of KA macaques. These data suggest that brain damage was confined to the ipsilateral hippocampus in the chronic period after KA-induced SRS in macaques.

### Hippocampal Volume and Neuropathology

Ipsilateral to the KA injection, hippocampal volumes were 1795±182 mm^3^. Cell counts were 26±10 neurons/100 µm^2^ and 18±5 glial cells/100 µm^2^ in the CA3 region of hippocampus. Hipocampal volumes ipsilateral to the KA injection show a positive correlation with the neuronal cells count in the CA3 (*P* = 0.02, r = 0.41); smaller volume were associated with a lower neuron cell count. Hippocampal volumes also correlate positively with the neuronal/glial ratio in the CA3 region (*P* = 0.005, r = 0.54). This means a smaller volume is associated with a lower ratio. The results are summarized in the Table S3.

## Discussion

Experimental animal models of mTLE are very helpful to elucidate the morphological and functional alterations of hippocampal sclerosis which are associated with epilepsy. Here we describe a new model of TLE induced by KA in macaques. Our main findings in this model include the following: (i) local, unilateral injection of KA in one dorsal hippocampus is able to induce SE in macaques, (ii) the SE followed by SRS associated with brain damage is mostly confined to the hippocampus, and (iii) hippocampal volume is in positive correlations with neuronal cells count, especially the ratio of neuron/glial cell in the CA3.

Our study demonstrates that neuropathological, imaging and electroclinical features representative of human mTLE can be reproduced in the macaque through local, unilateral injection of KA in one dorsal hippocampus. Our study method of acute SE induction had a very high efficiency without the acute mortality during local KA-induced SE in comparison to SE induced in the rodents by systemic treatment with either pilocarpine or KA. Furthermore, all KA-treated animals developed a late, chronic epileptic condition, characterized by spontaneous seizures. Partially, non-convulsive seizures were recorded during the chronic phase after a latent period that varied from 1 week to 3 months. In our model, seizures with mild motor features comparable to human mTLE seizures were observed. These events correlated with two main EEG-onset patterns described in the rat intrahippocampal KA model and in humans, that is, low-voltage fast activity and hypersynchronous patterns [29]–[31]. Detailed electrobehavioral studies conclude that seizures in chronic mTLE phase correlated with behavioral arrest, head nodding, and stereotyped behavior, such as exploration or grooming, without evident clonic or convulsive behavior [11], [14]. Our findings obtained on macaques indicate that spontaneous recurrent seizures in the chronic phase have no noticeable behavioral expression and require cortical ECOG recording to be identified. Unlike the macaques’ models, several studies reported that, in rats models, secondarily generalized convulsive seizures can be observed in chronic TLE phase after intrahippocampal KA injection [13], [32]. This discrepancy between the seizure features observed in rats and in macaques might not caused by the amount of KA utilized in the different protocols, but related to species-specific differences.

To investigate the effect of SRS on other associated brain regions, we performed a qualitative analysis of neuronal count and GFAP positive cells in several brain structures, including hilar regions, CA1, CA3, subiculum, temporal cortex, frontal cortex, entorhinal cortex, hypothalamus, thalamus and anterior hypothalamus as well. Epileptic macaques showed a marked cell loss in CA3, associated with GFAP staining score higher than score 2 in the ipsilateral hippocampus. And the contralateral hippocampus of the epileptic macaques or the ipsilateral hippocampus of the control macaque showed low GFAP and cell loss scores. Moreover, even if we recorded acute seizures in both hippocampus and other brain regions, the morphologic alterations described above were observed only in the hippocampus ipsilateral to KA injection, which suggested that the morphologic alteration was very likely due to the direct effects of KA activity, but not the secondary consequence of seizure.In line with the pathological findings, MRI performed in epileptic macaques showed that structural changes restricted to the hippocampus, similar to human mTLE. Our findings suggest that the brain damage seems to be restricted to the hippocampus during this chronic period. Similar to human mTLE, the sclerotic lesion is unilateral and restricted to the mesial region of one temporal lobe in our KA model. In contrast, in animal models induced by systemic application of either pilocarpine or KA, bilateral lesion of temporal lobes is a common finding and damage often involves extratemporal regions, such as thalamus and other olfactory and limbic cortices [4], [33]. Therefore, the brain damage restricted mainly to the hippocampus is the major differences between our models and the other non-human primates or rodents models [34]–[37].

In addition, macaques with SRS exhibited no cellular dispersion of neurons in the DG, which might be due to the small volumes (6∼8 µL) of KA injection in CA3 region. Also, our protocol induced a very restricted excitotoxic lesion limited to the site of KA injection. In line with this observation, no DG alteration moderate cell loss without granule cell dispersion or increased neurogenesis was found in more than 50% of patients with mTLE [38]. Our finding further confirmed that DG cellular dispersion may result from epilepsy-associated secondary changes, but not pathogenetically involved in the establishment of a chronic epileptic condition [38]. Moreover, a similar conclusion was reached by a study on the ratintrahippocampal KA model, in which dissociation between DG cell disperation and establishment of chronic mTLE was demonstrated [39].

In terms of soft tissues study, MRI displays an excellent resolution and is thus very useful in revealing morphological changes. Hippocampal sclerosis is characterized by increased hippocampal T2 signal and reduced hippocampal volume in MRI [40], [41]. Similarly, MRI performed in epileptic macaques demonstrated that 5 epileptic macaques had different degrees of hippocampal volume reduction and T_2_-weighted signal increase. However, only one epileptic macaque had hippocampal volume reduction without significant T_2_-weighted signal increase. The most convincing predictive element for the hippocampal volume is the extension and/or severity of hippocampal damage. Our study found that epileptic macaques showed a marked cell loss in CA3 associated with GFAP staining score higher than score 2 has a greater volumes reduction. Hippocampal volumes showed a positive correlation with the neuronal cells count in the CA3 region, especially with neuronal/glial ratio in the CA3 region; smaller volume was associated with a lower neuron cell count. However, hippocamplal volumes showed no significant correlation with the glial cell count in the CA3 region. Our results confirmed that hippocampal volume is best predicted by neuronal cell counts, especially ratio of neuron/glial cell. No significant T_2_ signal change of two epileptic macaques was observed in our experiments. Changes in T_2_-weighted images represent changes in free-water content within the brain; for that reason, any increase in signal is thought to reflect structural changes that disrupt water homeostasis, such as gliosis, edema, and neuronal loss. Previous report suggested a positive correlation between T_2_ weighted signal increase and gliosis [42], [43]. A high T2 signal was found mainly in the presence of severe gliosis, but not in moderate gliosis [43]. In line with this observation, a mild hippocampal gliosis was observed in the brain region with mild T_2_ signal change.

MRS is a noninvasive method that can provide indirect information about neuronal health, gliosis, energy metabolism, neuronal-glial cycling and molecular synthesis rates. In order to help us better understand the pathophysiological changes of focal epilepsy. NAA, which is found almost exclusively in neurons, has been considered to be a marker for neurons. Reduced NAA may be an early chemical marker of chronically epileptogenic brain tissue in humans and has been regarded as a marker of neuronal loss [44]. However, the Cho concentration is two to three folds higher in glial cells than in neurons [45]. The NAA/Cr and NAA/Cho ratio in the hippocampus may serve as a marker of gliosis [46], [47]. In this study, NAA, Cho and Cr measurements provided evidence for structural damage to the hippocampus in terms of neuronal loss and gliosis. NAA was shown to decrease in KA macaques compared with the control macaque. Neuronal loss was confirmed by H&E staining, which revealed the presence of damaged neurons in the CA3 region of hippocampus. Another key feature of endstage mesiotemporal sclerosis in human TLE, gliosis, was also identified in our KA macaques, as the increase in Cho-containing compounds and Cr are generally considered to be an indication of gliosis. The increased immunoreactivity of GFAP, a marker of reactive glia, further confirmed it.

As mentioned in the Introduction, this study evaluated the epileptogenesis and observed the behavioral manifestations, alteration of MRI&MRS imagings, histological pathology of the macaque epilepsy model. The typical neuropathologic, image and electroclinical features representative of human mTLE were observed in our models by acute pharmacologic treatment in an otherwise normal brain, mimics the patterns observed in human mTLE. Our study also raised the question that whether or not the mechanisms studied in the acute model of ictogenesis are representative of the chronic condition. Further study on the chronically epileptic brain with the method of vitro isolated macaque brain preparation might be required to answer this specific question. In summary, the present study is the first step in this direction and has confirmed that a chronic model of mTLE can be developed in the macaque.

## Conclusion

Our results suggest that a model of hippocampal mTLE can be developed in macaques by intrahippocampal injection of KA. Brain damage is confined to the hippocampus similar to human mTLE. The hippocampal volume correlates with the extension of the hippocampal damage (mainly due to neuronal cell loss and gliosis).

## Acknowledgments

We are grateful for the assistance of Dr. S.W. Li, Dr. Y. Fan and Dr. H.W Cheng in hippocampal volume measurement.

## Supporting Information

Figure S1 A comparison of hippocampal volumes between KA group (n = 6) and control group (n = 4).* ***P*** **<0.05**
(TIF)

Figure S2 **Frequency of SRS for all chronic animals distributed over time.** Macaque 1 to Macaque 6: KA group; Macaque 7 to Macaque 10: control group.
(TIF)

Table S1 **GFAP quantitative assessment for different brain regions.**
(DOCX)

Table S2 **Brain areas with neuronal damage evaluated by Nissl staining.**
(DOCX)

Table S3 **Simple correlations.**
(DOCX)
