# Supplementary figures and images for: A Macaque Model of Mesial Temporal Lobe Epilepsy Induced by Unilateral Intrahippocampal Injection of Kainic Acid
Source: PLoS One. 2013 Aug 26;8(8):e72336. doi: 10.1371/journal.pone.0072336 (PMC3753347; doi:10.1371/journal.pone.0072336)

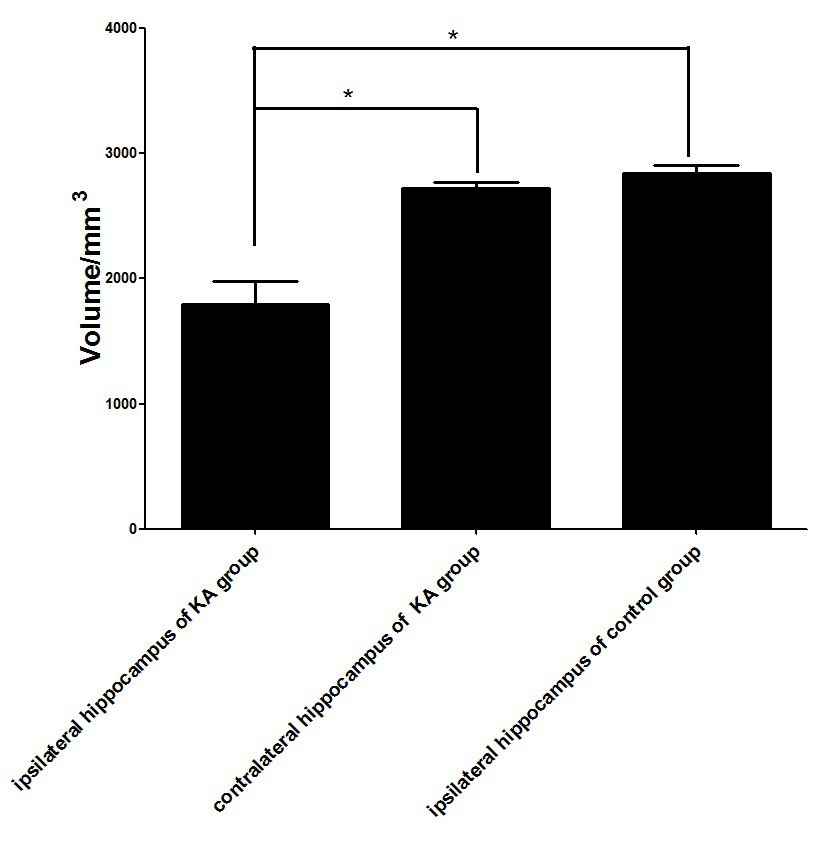

Supplement: Figure S1 — A comparison of hippocampal volumes between KA group (n = 6) and control group (n = 4).* P <0.05 (TIF) [file pone.0072336.s001.tif]

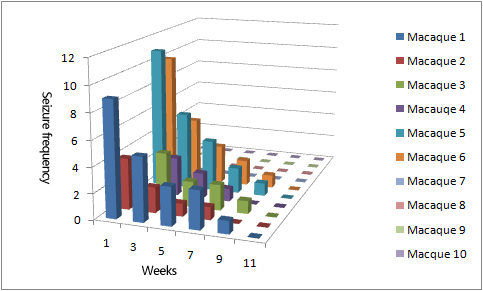

Supplement: Figure S2 — Frequency of SRS for all chronic animals distributed over time. Macaque 1 to Macaque 6: KA group; Macaque 7 to Macaque 10: control group. (TIF) [file pone.0072336.s002.tif]
