# Supplementary material for: A Macaque Model of Mesial Temporal Lobe Epilepsy Induced by Unilateral Intrahippocampal Injection of Kainic Acid
Source: PLoS One. 2013 Aug 26;8(8):e72336. doi: 10.1371/journal.pone.0072336 (PMC3753347; doi:10.1371/journal.pone.0072336)
Supplement: Table S1 — GFAP quantitative assessment for different brain regions. (DOCX) [file pone.0072336.s003.docx]

**Table.S1** GFAP quantitative assessment for different brain regions.

| Regions | Control | KA | |
| --- | --- | --- | --- |
|  | Ipsilateral | Contralateral | Ipsilateral |
|  | (n=4) | (n=6) | (n=6) |
| Hilus | 0 | 0 | 0 |
| CA1 | 0 | 0 | 0 |
| CA3 | 0 | 0 | 2.67±0.52 |
| Entorhinal cortex | 0 | 0 | 0 |
| Temporal cortex | 0 | 0 | 0 |
| Frontal cortex | 0 | 0 | 0 |
| Thalamus | 0 | 0 | 0 |
| Hypothalamus | 0 | 0 | 0 |
| Anterior Hypothalamus | 0 | 0 | 0 |

For GFAP-stained sections were quantitatively assessed as follows: score 0- no obvious expression, score 1- obvious expression involving 10-25% of the region; score 2- obvious expression involving 40-60% of the region; score 3- >80% the expression in the regions of interest. Assessment was performed in hilar regions, CA1, CA3, subiculum, temporal cortex, frontal cortex, entorhinal cortex, hypothalamus, thalamus and anterior hypothalamus in per animal.
