# Supplementary material for: A Macaque Model of Mesial Temporal Lobe Epilepsy Induced by Unilateral Intrahippocampal Injection of Kainic Acid
Source: PLoS One. 2013 Aug 26;8(8):e72336. doi: 10.1371/journal.pone.0072336 (PMC3753347; doi:10.1371/journal.pone.0072336)
Supplement: Table S2 — Brain areas with neuronal damage evaluated by Nissl staining. (DOCX) [file pone.0072336.s004.docx]

**Table.S2** Brain areas with neuronal damage evaluated by Nissl staining.

| Regions | Control | KA | |
| --- | --- | --- | --- |
|  | Ipsilateral | Contralateral | Ipsilateral |
|  | (n=4) | (n=6) | (n=6) |
| Hilus | 0 | 0 | 0 |
| CA1 | 0 | 0 | 0 |
| CA3 | 0 | 0 | 2.75±0.42 |
| Entorhinal cortex | 0 | 0 | 0 |
| Temporal cortex | 0 | 0 | 0 |
| Frontal cortex | 0 | 0 | 0 |
| Thalamus | 0 | 0 | 0 |
| Hypothalamus | 0 | 0 | 0 |
| Anterior Hypothalami | 0 | 0 | 0 |

The severity of neuronal damage in various brain regions was scored as follows: score 0, no obvious damage; score1, apparent alteration of morphology, but no unambiguous lesion; score 2, clear-cut lesions involving 20–50% of neurons; and score 3, clear-cut lesions involving >50% of neurons. Assessment was performed in hilar regions, CA1, CA3, subiculum, temporal cortex, frontal cortex, entorhinal cortex, hypothalamus, thalamus and anterior hypothalamus in per animal.
