# Supplementary material for: A Macaque Model of Mesial Temporal Lobe Epilepsy Induced by Unilateral Intrahippocampal Injection of Kainic Acid
Source: PLoS One. 2013 Aug 26;8(8):e72336. doi: 10.1371/journal.pone.0072336 (PMC3753347; doi:10.1371/journal.pone.0072336)
Supplement: Table S3 — Simple correlations. (DOCX) [file pone.0072336.s005.docx]

**Table.S3** Simple correlations

| Dependent variable | Independent variable | P Value | R Value | Slope |
| --- | --- | --- | --- | --- |
| Hippocampal volume | Neuron count CA3 | 0.02 | 0.41 | Positive |
|  | Glial count CA3 | 0.293 |  |  |
|  | Ratio of Neuron/Glial | 0.005 | 0.54 | Positive |

MRI measurements on the ipsilateral hippocampus of the KA injection. A positive correlation indicates that a higher value of the dependent variable is associated with a higher value of the independent variable. P<0.05 was considered statistically significant.
